# Multivariable Linear Models Outperform 2^−ΔΔCT^ for qPCR Data Analysis

**DOI:** 10.21203/rs.3.rs-6014969/v1

**Published:** 2025-02-13

**Authors:** Thomas H. Hampton, Lily Taub, Kiyoshi Ferreria-Fukutani, Bruce A. Stanton, Todd A. MacKenzie

**Affiliations:** Geisel School of Medicine at Dartmouth; Geisel School of Medicine at Dartmouth; Geisel School of Medicine at Dartmouth; Geisel School of Medicine at Dartmouth; Geisel School of Medicine at Dartmouth

**Keywords:** qPCR, delta-delta CT, amplification efficiency

## Abstract

Here we present a method based on multivariable linear models for qPCR data analysis as an alternative to the most commonly used method, 2^−ΔΔCT^. It has long been understood that amplification efficiency during qPCR may be less than two, that is, the amount of DNA present may not double in each cycle, and it is also known that amplification efficiency may differ between target and reference genes. Therefore, it has long been recommended that qPCR experiments include direct assessment of amplification efficiency, and that efficiency values be included in the calculation of differential gene expression. Nonetheless, current reports that include qPCR data continue to use 2^−ΔΔCT^, even though 2^−ΔΔCT^ assumes an efficiency of two in both reference and target genes. Using multivariable linear models for qPCR data analysis does not require direct measurement of amplification efficiency but provides correct significance estimates for differential expression even when amplification is less than two or differs between target and reference genes. We introduce the logic behind using multivariable linear models in the context of qPCR data analysis, the mathematics behind using them, and provide simulations demonstrating that multivariable linear models outperform 2^−ΔΔCT^ for qPCR data analysis.

## Introduction

Throughout this report, we will make use of data from one of our recently published studies to illustrate the utility of multivariable linear models for the analysis of qPCR cycle threshold (CT) data. In that study, we exposed primary human airway epithelial cells from five cystic fibrosis (CF) donors to ETI (elexacaftor, tezacaftor and Ivacaftor) or vehicle (DMSO) for 48 hours^1^. ETI is well tolerated and vastly improves lung function in people with cystic brosis^2,3^. Cycle thresholds were calculated for several target genes (*DEFB1, MMP10, MMP12, IL1B, TNF*) and reference genes (*HSP90AB, GAPDH, HPRT1, GUSB* and *UBC*). In that paper, we used ANCOVA, a kind of multivariable linear model (MLM) to analyze our qPCR results because ANCOVA was more consistent with the statistical approaches we used to analyze RNA-seq and proteomic results than a classic 2^−ΔΔCT^ approach^4^ would have been. 2^−ΔΔCT^ remains highly popular^5^ despite well documented technical limitations^6^. As will be described in greater detail, the main difference between 2^ΔΔCT^ and more robust qPCR data analysis approaches is that the latter incorporate amplification efficiency estimates of target and references genes into their calculations whereas 2^−ΔΔCT^ assumes that amplification efficiency of both genes is 2. Our simulations confirm that using analysis of covariance (ANCOVA) for qPCR data analysis represents a sensible compromise between the added effort of measuring amplification efficiency, e.g., by running a sample dilution curve, and legitimate concerns that failure to address amplification efficiency might compromise experimental results.

### qPCR remains popular but following data analysis best practices is not

**Figure 1:**
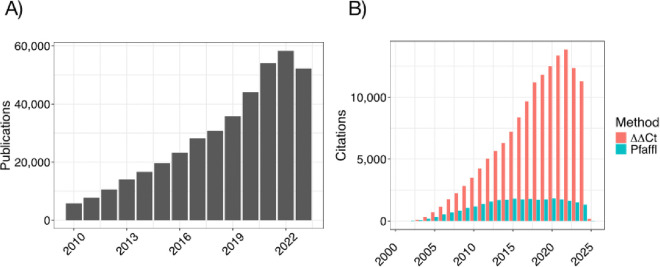
A) Number of PubMed Central publications mentioning “qPCR” in the Methods section since 2000. B) PubMed Central citations since 2000 for the original 2^−ΔΔCT^ paper by Livak and Schmittgen^4^ and the seminal Pfaffl paper advocating for correcting CT values for amplification efficiency^6^.

Papers mentioning qPCR in their methods section have grown steadily throughout the 21st century (Fig. 1A) as have citations of the original 2^−ΔΔCT^ paper^4^ but citations of the highly recommended Pfa method that incorporates amplification efficiency into its calculations^6^ have not (Fig. 1B). Our survey of 20 recent publications in PubMed Central containing “qPCR” as a methods keyword (Table 1) replicates findings reported by Bustin et al.^5^ suggesting that about 75% of published qPCR results use the 2^−ΔΔCT^ method and fewer than 5% explicitly take amplification efficiency into account.

The disconnect between what the qPCR community has been advised to do and what the community actually does is significant, but we think the simplicity and statistical efficiency of using multivariable linear models (MLM) for qPCR make it an appealing alternative to 2^−ΔΔCT^.

### ANCOVA models of qPCR compared to 2^−ΔΔCT^

In this section, we compare the commonly used 2^−ΔΔCT^ approach to a multivariable linear model like ANCOVA for qPCR data analysis. For our data set both approaches yielded comparable results but the mathematical properties of ANCOVA may make it superior in general. To understand why, we will explore how each approach works. The mathematical basis for using ANCOVA in this context of qPCR can be found in the Appendix.

2^−ΔΔCT^ uses two levels of control, a control for the treatment, e.g. treatment vs control, and a control for the quality of the sample, namely the reference gene. The practice of raising 2 to the difference of cycle thresholds (CT) reflects that during each round of amplification in qPCR, the amount of material should roughly double. Raw CT values are therefore naturally on a log base 2 scale and suitably distributed for tests that assume normal distribution. 2^−ΔΔCT^ calculates the average difference between the target gene CT and the reference gene CT in the experimental groups and control groups. Next, the difference in these averages is typically calculated and compared using a t-test or paired t-test. Alternatively, the distribution of the target and reference gene differences can be compared between the exposed and control samples using a rank-based approach. Users may choose to “back-transform” the average difference of the differences by raising 2 to the difference in CT. In that case, because CT is inversely related to the amount of starting material, an intuitively meaningful back-transformation incorporates a minus sign in the exponent: 2^−ΔΔCT^. However, remaining on the log scale throughout can avoid possible statistical problems involved with back transformation^9^.

The reasoning underlying 2^−ΔΔCT^ is *difference in differences;* a ubiquitous study design in economics, social sciences and epidemiology^10^. For example, in economics it is common to evaluate the effect of a policy, such as introduction of a new therapy into Medicare or Medicaid, by comparing results between states that had the policy change and states that did not have the policy change, before and after the introduction of the policy. An example of difference-in-differences in clinical research is the randomization of patients into two groups, a treatment group and control group, with measurements of the dependent variable at baseline and post-baseline.

**Figure 2:**
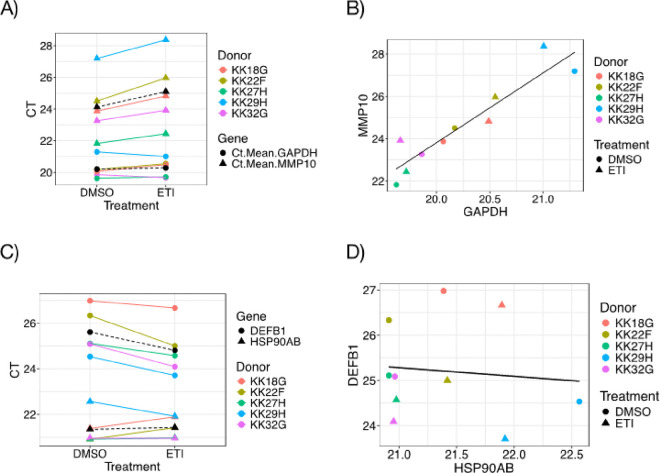
A) Mean CT values for triplicate measurements *GAPDH* (circles) and *MMP10* (triangles) in samples from 5 donors (colors) exposed to DMSO or ETI. Colored lines connect samples from the same donor. Black symbols and dashed lines represent donor averages for each gene and treatment. B) *MMP10* CT values as a function of *GAPDH* CT value for each of the 10 observations in Figure 2A. C) Mean CT values for triplicate measurements *HSP90AB (*circles) and *DEFB1* (triangles) in samples from 5 donors (colors) exposed to DMSO or ETI. Colored lines connect samples from the same donor. Black symbols and dashed lines represent donor averages for each gene and treatment. D) *DEFB1* CT values as a function of *HSP90AB*CT value for each of the 10 observations in Figure 2C.

In most difference-in-difference designs the dependent variable is the same, for instance, one might use mortality rate as the dependent variable in different states, before and after a policy change. 2^−ΔΔCT^ does not use the same dependent variable: CT for the target gene and reference gene are independent. Consequently, these two measures may have little or no correlation, and may have vastly different variation. Mathematically, 2^−ΔΔCT^ assumes that sample quality affects the value of the target gene and the reference gene by the same amount. However, it is possible, and even likely, that sample quality and other factors such as primer design and cycling conditions affect reference and target genes in different amounts. For instance, it may be that if sample quality and/or primer design and cycling conditions are impacting the reference gene by x, they impact the target gene by the amount k*x, where k is some number. 2^−ΔΔCT^ assumes that k = 1. A value of zero would indicate the target gene has no ability to inform on the quality of the sample whereas a statistically significant negative value would be hard to interpret biologically and would suggest an error. One advantage of multivariable linear models (e.g., ANCOVA) is that they control for variation due to sample quality and cycling conditions to the extent that the reference gene reflects that variability. If the reference gene is not capturing sample quality and cycling conditions, a multivariable linear model will essentially ignore it. The ability of the reference gene to capture variation in sample quality and cycling conditions can also be assessed using the related method of Pearson or Spearman (rank based) correlations applied to the reference and target genes as presented in Fig. 2B and 2D. If there is no correlation between the target and reference genes, the 2^−ΔΔCT^ method is dubious, and subtracting the reference gene CT from the target gene CT actually reduces the power of the study. Coefficients different from k = 1 can also account for amplification efficiency differences between target and reference genes, making ANCOVA significantly more robust than 2^−ΔΔCT^ when amplification efficiency differences are an issue.

In summary, ANCOVA models of qPCR use a reference to control for differences in sample quality. However, instead of simply subtracting reference values from the gene of interest values, ANCOVA uses regression to establish the level of correction to apply. Applying ANCOVA to qPCR data requires fewer steps than using 2^−ΔΔCT^ to normalize results and perform a statistical test: ANCOVA uses a reference to account for sample quality variability and assesses the significance in one step.

For example, here are *GAPDH* and *MMP10* CT values from our recent study^1^ (Table 2).

An ANCOVA analysis of the data in Table 2 requires just two lines of R. The first line says: “Use the data in Table 2.data to estimate the impact of Donor, Treatment and *GAPDH* on *MMP10* and store the result in a variable called fit.” The second line displays the result.

fit<-lm(MMP10~Donor+Treatment+GAPDH,data=Table2.data)summary(fit)

Key estimates and p-values generated by this ANCOVA model are shown in Table 3. The second to last row includes the term “Treatment-ETI.” The name of the term includes a factor name “Treatment” and the name of the level “ETI.” The estimate for Treatment ETI is an increase of about 1 CT (0.944) compared to Treatment DMSO. This squares with Fig. 2A: exposure to ETI raised CT by roughly 1 unit in each donor, and it was significant (p = 0.013).

The last row in Table 3 shows that *GAPDH* is not explaining a statistically significant amount of variation in the target gene (CT = 0.352, p = 0.595), but the estimate has the same (positive) sign as the treatment effect, which is what we expect.

It is easy to apply ANCOVA analysis to data structured like Table 2. For example, the following R command estimates the impact and significance of Donor, Treatment and *HSP90AB* on *DEFFB1* CT values using data from a table called All.CT:

lm(DEFB1~Donor+Treatment+HSP90AB,data=All.CT)

This command produced the estimates and significance shown in Table 3:

Table 3 shows that ETI significantly reduces *DEFB1* CT consistent with Fig. 2C, and that *HSP90AB* does not itself respond to ETI (estimate = 0.011 CT, p = 0.984). Figure 2D shows that *HSP90AB* and *DEFB1* are uncorrelated. This is an example of a situation where simply subtracting CT values of the reference gene from the gene of interest, which is what 2^−ΔΔCT^ would do, would yield inferior results compared to ANCOVA.

### ANCOVA handles reaction efficiency differences between target and reference gene

The ANCOVA multivariable linear model offers another advantage over the 2^−ΔΔCT^ approach. Unlike 2^−ΔΔCT^ it is invariant to any difference in reaction efficiency between the target and reference genes. Let $e_{T}$ (a number between 0 or 1, i.e. 0–100%) be the reaction efficiency of the target gene. In general, we should be using the logarithm with base $1+e_{T}$. Using the change of base property of logarithms, ${log}_{b}c={log}_{a}c^{*}{log}_{b}a$, the cycle thresholds calculation is off by a factor ${log}_{2}1+e_{T}$ Similarly, if $e_{R}$ is the reaction efficiency of the reference gene its cycle thresholds will be off by the factor ${log}_{2}1+e_{R}$. Fortunately, it is a property of multivariable linear models that the p-values are invariant to scale changes. That is, any difference in reaction efficiency (i.e., cycling conditions) between the target and reference gene will not change the p-values from the multivariable linear model we propose.

### Simulations validate multivariable models like ANCOVA for qPCR

Although our published paper successfully applied a MLM to qPCR data analysis^1^, we ran simulations to establish that MLM would give unbiased results for typical qPCR applications with small sample sizes whose distributions may differ somewhat from the normal distributions linear models assume. As detailed below, simulations suggest that ANCOVA works well for qPCR and may be more powerful in practice than 2^−ΔΔCT^. Simulations were also used to assess the impact of correlation between target and reference genes (Table 4) and what happens when the reference gene responds to treatment effects (Table 5).

Rules of thumb for the sample size necessary to conduct a linear regression, e.g. ANCOVA, range from at least two^11^ to up to eight observations^12^ for every paramter estimated (e.g. variable) in your model. If this ratio of sample size to parameters estimated is too small then the p-values reported are less reliable. This is becuse the p-values from linear regression, like the p-values from two sample t-tests, assume that the underlying distribution is Gaussian (e.g. bell shaped). The p-values reported may be biased toward accepting or rejecting the null hypothesis when the underlying distribution is not Gaussian. Ideally, one should reject the null hypothesis about 5% of the time when the null hypothesis is true, e.g., when there is no true difference between experimental groups. Rejecting the null hypothesis less than 5% of the time when there is no true difference suggests that a test may too conservative. Tests that are too conservative, reduce power and inflate Type II errors. The flip side, rejecting the null hypothesis more than 5% of the time when there is no true difference suggests that a test is too liberal and inflates Type I errors. In our application there are three degrees of freedom, one for the treatment, one for the reference gene and one for the intercept, as well as one for the random intercept we used. This is four degrees of freedom (parameters to estimate) whereas our sample size is ten: just enough, because ten is greater than two times four.

We assessed: (i) any bias in the rejection rate of the multivariable linear model (MLM) in comparison to 2^ΔΔCT^, as well as ΔCT (which does not use a reference gene), and (ii) the power of MLM in comparison to 2^ΔΔCT^ and ΔCT. We evaluated the performance of the MLM when the underlying distribution is right skewed and left skewed. We consider the scenario when there are a total of 10 observations (e.g. 10 donors) in which 5 are treated and 5 are controls. To compare how MLM and 2^−ΔΔCT^ perform in terms of the ability of the reference gene to capture sample quality, we evaluated performance with respect to the correlation of the target and reference gene; specifically, we considered a very strong Pearson correlation of 0.9, a moderate correlation of 0.5 and zero correlation. The code used in the simulation is included in GitHub (https://github.com/DartCF/MLM-for-qPCR).

As shown in Table 4, the empirical type I error rate of the MLM approach is close to the nominal value (5%) when the distribution is not Gaussian (right skewed, left skewed) for each of the three correlation parameters (0, 0.5 and 0.9). This indicates that the multivariable approach is a statistically valid approach (e.g. little or no bias in Type I error). MLM showed slight bias in the conservative direction, that is, empirical Type I error rate was a bit less than 5%. 2^−ΔΔCT^ was more conservative in every scenario, in other words, less powerful than MLM.

Table 4 illustrates the ability of the MLM to control for the reference gene to the extent that it is relevant. When there is very little shared variation in sample quality or cycling conditions between the target and reference genes, their correlation is close zero, reducing the power of 2^−ΔΔCT^ in comparison to the best approach in that case, ΔCT (not using a reference gene at all). However, MLM has almost the power of ΔCT, even when the distribution is Gaussian or right skewed or left skewed. When there is a high correlation (e.g., 0.9), 2^−ΔΔCT^ outperforms ΔCT (100% vs 78.8% for Gaussian) but MLM is just as good (100%). When there is moderate correlation of the target and reference gene, MLM outperforms both 2^−^ΔΔCT and ΔCT.

Table 5 shows the results of simulations when the treatment has an effect on the reference gene equal to one standard deviation. This is a big problem. In this setting, only ΔCT (ignoring the reference gene) is a valid approach. 2^−ΔΔCT^ is invalid when the exposure affects the reference gene, because it will identify an effect on the target gene in the opposite direction. For instance, if the correlation of the target and reference gene is high, the 2^−ΔΔCT^ falsely rejects the null hypothesis with a frequency of 93%. MLM is also invalid in this setting unless there is zero correlation between target and reference gene.

### Limitations

Both ANCOVA and 2^−ΔΔCT^ assume reference gene stability. Although ANCOVA models offer advantages over 2^−ΔΔCT^, including offering greater statistical power and handling variable amplification efficiency better than 2^−ΔΔCT^, effect estimates provided by ANCOVA models are in CT units, not fold change. CT units are only equal to 1 log2 unit of change in the special case where amplification efficiency is 100%.

## Discussion

qPCR remains a foundational technique in molecular biology, with about 5,000 new citations every month (Fig. 1A). Most practitioners control for possible variability using a single, well-known reference gene such as *GAPDH* without explaining how this gene was chosen or providing evidence of its stability^5^. This practice could obscure true treatment effects^13^, degrading reproducibility. Most researchers assess qPCR differential gene expression using the 2^−ΔΔCT^ method, and therefore tacitly assume perfect amplification efficiency, potentially distorting findings^6^, but it is impossible to know because researchers rarely share raw data^14^. Solutions to many reproducibility problems have been proposed and reviewed^15^ but largely ignored^5^ perhaps because validating reference genes, performing dilution curves to measure amplification efficiency and adhering to data standards is viewed as a low priority by study authors and peer reviewers.

If the precise magnitude of fold change were important to the research question, running a sample dilution curve could establish efficiency, and those estimates could be used to create efficiency-weighted CT values suitable for downstream statistical analysis^16^. Often, what matters most is whether a treatment significantly affects gene expression in the statistical sense (p < .05) and our simulations show that multivariable linear models like ANCOVA are superior to 2^−ΔΔCT^. Using ANCOVA for qPCR data analysis requires very little coding and provides p-values that are invariant to amplification efficiency differences between the reference and gene of interest. Moreover, because ANCOVA can be more sensitive than 2^ΔΔCT^, ANCOVA will sometimes detect experimental differences with fewer samples, saving time and resources. Multivariable linear models naturally accommodate the more complex experimental designs that are increasingly common in science. Based on simulations, ANCOVA offers a lower false negative rate than 2^ΔΔCT^, especially when the reference gene does not provide useful information about the gene of interest.

## Figures and Tables

**Table 1:** Survey of 20 recent papers mentioning qPCR in the text of Methods section, including PubMed Central ID, Method Ref (whether the paper cited a method for qPCR data analysis), Norm Type (type of normalization used, if stated), Reference Approach (reference genes used, if stated) and in the last column, whether amplification efficiency was used ^5 , 8^ .

ID	Method Ref	Norm Type	Reference Approach	Efficiency Reported
PMC11645144	No	2^−ΔΔCT^		No
PMC11567002	No	2^−ΔΔCT^	*GAPDH, ACTB*	No
PMC11567001	No		*GAPDH, ACTB*	No
PMC11566997	No			No
PMC11559625	No	Standard Curve	Mean of 2 genes	No
PMC11554307	No		*ACTB*	No
PMC11554301	No	Standard Curve	*ACTB*	No
PMC11546848	No		*rp49*	No
PMC11542633	No	2^−ΔΔCT^	*S7 rRNA*	No
PMC11540302	No			No
PMC11540031	No		*ACT7*	No
PMC11566318	Yes	Pfaffl	*ACTB*	No
PMC11527450	No	2^−ΔΔCT^	*ACTB*	No
PMC11527446	No	2^−ΔΔCT^	*B2M*	No
PMC11537643	No	2^−ΔΔCT^	*GAPDH*	No
PMC11537631	No	2^−ΔΔCT^	*GAPDH*	No
PMC11529709	Yes	QBase^7^		No
PMC11567019	No		*GAPDH*	No
PMC11529476	No		*GAPDH*	No
PMC11554380	Yes	2^−ΔΔCT^	*GAPDH*	No

**Table 2:** Sample annotations (Donor, Treatment, Group) and CT values for *GAPDH* and MMP70 from Fig. 2.

Donor	Treatment	Group	*GAPDH*	*MMP10*
KK22F	DMSO	KK22F DMSO	20.17	24.50
KK22F	ETI	KK22F ETI	20.55	25.96
KK32G	DMSO	KK32G DMSO	19.86	23.26
KK32G	ETI	KK32G ETI	19.66	23.92
KK27H	DMSO	KK27H DMSO	19.62	21.82
KK27H	ETI	KK27H ETI	19.72	22.44
KK29H	DMSO	KK29H DMSO	21.29	27.19
KK29H	ETI	KK29H ETI	21.01	28.37
KK18G	DMSO	KK18G DMSO	20.06	23.87
KK18G	ETI	KK18G ETI	20.49	24.82

**Table 3:** ANCOVA analysis of the data in Table 2.*MMP10* CT were predicted as a functionof Donor, Treatment and *GAPDH*. ETItreatment significantly raises CT by0.944 (p = 0.013), as shown in thesecond to last row.

Term	Estimate	p_value
Donor KK22F	0.859	0.054
Donor KK27H	−1.998	0.021
Donor KK29H	3.127	0.013
Donor KK32G	−0.574	0.256
Treatment-ETI	0.944	0.013
*GAPDH*	0.352	0.595

**Table 3:** ANCOVA analysis of the data in ALL.CT.*DEFB1* CT were predicted as a functionof Donor, Treatment and *HSP90AB.* ETITreatment significantly raises CT by-0.803 (p = 0.031), as shown in thesecond to last row.

Term	Estimate	p_value
DonorKK22F	−1.150	0.063
DonorKK27H	−1.973	0.024
DonorKK29H	−2.709	0.008
DonorKK32G	−2.225	0.017
Treatment-ETI	−0.803	0.031
HSP90AB	0.011	0.984

**Table 4:** **(Left)** Empirical Type I Error Rate for each model (MLM, 2^−ΔΔCT^, ΔCT). Correlation between target and reference gene, distribution type (Gaussian, Right Skewed, Left Skewed). **(Right)** Power for each model shown at left.

		Empirical Type I Error Rate			Power		
Correlation	Distribution	MLM	2^−ΔΔCT^	ΔCT	MLM	2^−ΔΔCT^	ΔCT
**0**	**Gaussian**	5.0%	4.5%	4.2%	71.5%	47.6%	76.4%
**0.5**	**Gaussian**	4.8%	4.1%	4.2%	92.7%	85.5%	85.7%
**0.9**	**Gaussian**	5.0%	4.7%	4.8%	100.0%	100.0%	78.8%
**0**	**Right Skewed**	4.4%	3.7%	2.8%	76.1%	52.9%	78.3%
**0.5**	**Right Skewed**	4.2%	3.4%	4.0%	91.9%	84.0%	84.9%
**0.9**	**Right Skewed**	4.7%	3.9%	4.4%	99.9%	99.9%	79.4%
**0**	**Left Skewed**	4.2%	3.5%	2.8%	75.3%	52.8%	78.1%
**0.5**	**Left Skewed**	4.6%	3.7%	4.2%	91.8%	84.2%	85.5%
**0.9**	**Left Skewed**	4.3%	3.5%	4.2%	99.9%	99.9%	78.8%

**Table 5:** **(Left)** Empirical Type I Error Rate for each model (MLM, 2^−ΔΔCT^, ΔCT). Correlation between target and reference gene, distribution type (Gaussian, Right Skewed, Left Skewed) **(Right)** Power for each model shown at left.

		Empirical Type 1 Error Rate			Power		
Correlation	Distribution	MLM	2^−ΔΔCT^	ΔCT	MLM	2^−ΔΔCT^	ΔCT
**0**	**Gaussian**	5.0%	14.9%	4.8%	63.2%	15.6%	77.1%
**0.5**	**Gaussian**	12.7%	32.9%	4.1%	63.6%	32.9%	85.6%
**0.9**	**Gaussian**	75.5%	93.2%	4.4%	88.2%	93.0%	78.4%
**0**	**Right Skewed**	4.5%	18.2%	2.8%	65.1%	18.1%	78.5%
**0.5**	**Right Skewed**	17.6%	37.9%	4.2%	65.0%	37.7%	85.9%
**0.9**	**Right Skewed**	77.0%	91.0%	4.3%	88.2%	89.6%	78.9%
**0**	**Left Skewed**	4.9%	17.8%	2.7%	65.9%	17.7%	79.0%
**0.5**	**Left Skewed**	17.2%	37.6%	4.2%	65.2%	37.9%	85.8%
**0.9**	**Left Skewed**	77.1%	90.7%	4.6%	88.7%	90.8%	78.7%

## Data Availability

The code used in the simulation is included in GitHub (https://github.com/DartCF/MLM-for-qPCR).
